# Genetic risk for Alzheimer’s disease is concentrated in specific macrophage and microglial transcriptional networks

**DOI:** 10.1186/s13073-018-0523-8

**Published:** 2018-02-26

**Authors:** Katherine E. Tansey, Darren Cameron, Matthew J. Hill

**Affiliations:** 1 Core Bioinformatics and Statistics Team, College of Biomedical and Life Sciences, Cardiff University, Cardiff, UK; 2 MRC Centre for Neuropsychiatric Genetics and Genomics, Division of Psychological Medicine and Clinical Neurosciences, School of Medicine, College of Biomedical and Life Sciences, Cardiff University, Hadyn Ellis Building, Maindy Road, Cardiff, CF24 4HQ UK; 3 UK Dementia Research Institute, Cardiff University, Hadyn Ellis Building, Maindy Road, Cardiff, CF24 4HQ UK

**Keywords:** Alzheimer’s disease, GWAS, Transcription factor, Non-coding variation

## Abstract

**Background:**

Genome-wide association studies of Alzheimer’s disease (AD) have identified a number of significant risk loci, the majority of which lie in non-coding regions of the genome. The lack of causal alleles and considerable polygenicity remains a significant barrier to translation into mechanistic understanding. This includes identifying causal variants and the cell/tissue types in which they operate. A fuller understanding of the cell types and transcriptional networks involved in AD genetic risk mechanisms will provide important insights into pathogenesis.

**Methods:**

We assessed the significance of the overlap between genome-wide significant AD risk variants and sites of open chromatin from data sets representing diverse tissue types. We then focussed on macrophages and microglia to investigate the role of open chromatin sites containing motifs for specific transcription factors. Partitioned heritability using LDscore regression was used to investigate the contribution of specific macrophage and microglia transcription factor motif-containing open chromatin sites to the heritability of AD.

**Results:**

AD risk single nucleotide polymorphisms (SNPs) are preferentially located at sites of open chromatin in immune cells, particularly monocytes (*z* score = 4.43; corrected *P* = 5.88 × 10^− 3^). Similar enrichments are observed for macrophages (*z* score = 4.10; corrected *P* < 2.40 × 10^− 3^) and microglia (*z* score = 4.34, corrected *P* = 0.011). In both macrophages and microglia, AD risk variants are enriched at a subset of open chromatin sites that contain DNA binding motifs for specific transcription factors, e.g. SPI1 and MEF2. Genetic variation at many of these motif-containing sites also mediate a substantial proportion of AD heritability, with SPI1-containing sites capturing the majority of the common variant SNP-chip heritability (microglia enrichment = 16.28, corrected enrichment *P* = 0.0044).

**Conclusions:**

AD risk alleles plausibly operate in immune cells, including microglia, and are concentrated in specific transcriptional networks. Combined with primary genetic association results, the SPI1 and MEF2 transcriptional networks appear central to AD risk mechanisms. Investigation of transcription factors targeting AD risk SNP associated regulatory elements could provide powerful insights into the molecular processes affected by AD polygenic risk. More broadly, our findings support a model of polygenic disease risk that arises from variants located in specific transcriptional networks.

**Electronic supplementary material:**

The online version of this article (10.1186/s13073-018-0523-8) contains supplementary material, which is available to authorized users.

## Background

Genome-wide association studies (GWAS) of Alzheimer’s disease (AD) have identified multiple loci containing common variant risk alleles [1]. These findings offer new routes to understanding disease biology that could be used to design novel therapies. However, like other complex diseases and traits, the majority of these risk alleles are located in non-coding regions of the genome [2], making immediate functional interpretation difficult. Furthermore, at each locus the risk signal is often associated with multiple variants in strong linkage disequilibrium (LD), any of which could credibly be the causal variant(s). Nevertheless, analytical approaches, such as pathway analysis [3] and integration with chromatin annotations [4, 5], have begun to identify the cell types and processes that are likely to be disrupted by AD risk alleles. Strikingly, these complementary approaches have identified immune cells and pathways as the likely effectors of AD genetic risk. Despite these advances, the full repertoire of potentially causal cell types and the molecular mechanisms through which AD risk variants operate have yet to be fully investigated. This includes the identification of functional variants at genome-wide significant risk loci as well as the mechanisms through which polygenic risk operates.

Of these approaches, integration of genetic association data with the growing amount of functional genomic annotations (e.g. ENCODE [6] and Roadmap Epigenomics [7]) have the potential to identify: (1) causal non-coding risk alleles, (2) the mechanisms by which they operate and (3) the cell types in which they function [8, 9]. While risk alleles at genome-wide significant loci represent robust findings suitable for biological characterisation, it is now known that thousands of variants throughout the genome contribute to disease heritability [10]. Recently developed analytical methods, such as stratified LDscore regression [11, 12], can use these annotations to investigate the relevance of specific cell types to the heritability of a disease of interest, extending analysis beyond genome-wide significant loci to capture polygenic risk mechanisms.

Several technologies now exist for genome-wide identification of non-coding elements with regulatory potential. These range from the study of post-translational modifications of histones to the resolution of binding sites for specific transcription factors; collectively termed chromatin immunoprecipitation (ChIP). Methods that rely on discriminating local chromatin structure, such as DNase-seq [13] and assay for transposase-accessible chromatin using sequencing (ATAC-seq) [14], can identify potential transcription factor binding sites without the need for performing multiple transcription factor ChIP experiments. These open chromatin regions (OCRs) display a high degree of cell-type specificity, defining promoters of expressed genes as well as distal regulatory elements [13], and are enriched for DNA motifs recognised by transcription factors important for determining cell lineage and function [15]. Although the integration of chromatin annotations with GWAS results has been successful in identifying disease-relevant tissues [2, 8, 16], few, if any, have attempted to attribute genome-wide polygenic risk mechanisms to specific transcription factor networks.

We, therefore, reasoned that the integration of results from GWAS of AD with OCRs from multiple cell types would pinpoint disease relevant cell types and link AD genetic risk variants to specific transcriptional networks active in those cell types.

## Methods

### Data processing

DNase hypersensitivity sites (DHSs) and histone ChIP-seq peaks (H3K4me3, H3K4me1 and H3K27ac) were generated by the Roadmap Epigenomics Project [7]. Monocyte and macrophage DNase-seq data were generated by Blueprint (http://dcc.blueprint-epigenome.eu/#/home). All data sets had been mapped to hg19 (GRCh37). Data were processed using BEDTools [17]. Cancer-derived cells lines present in the Epigenomics Roadmap data set were removed before further analyses. Microglia ATAC-seq data [18] were obtained from dbGaP Study Accession: phs001373.v1.p1. Data were aligned to hg19 (GRCh37) using bwa [19] and peaks were called using hotspot [20], following the protocol described by the Blueprint Consortium.

### Enrichment testing for the overlap between AD risk variants and open chromatin regions

Genome-wide significant (*P* < 5 × 10^− 8^) AD risk variants [(GWAS index single nucleotide polymorphisms (SNPs)] identified by Lambert et al. [1] were downloaded from the GWAS catalogue [21]. Variants located in the APOE and major histocompatibility complex (MHC) regions were excluded, resulting in 18 GWAS index SNPs. For the remaining GWAS index SNPs, 10,000 matched sets of variants were generated using SNPsnap [22], which matches SNPs based on allele frequency, number of SNPs in LD, distance to nearest gene and gene density. Variants in high LD (*r*^2^ > 0.8) with each SNP (GWAS index SNPs and matched sets) were extracted from the 1000 Genomes Project (phase 3). The resulting 10,001 SNP sets were then intersected with OCRs and histone peaks using BEDTools. The number of overlapping loci was calculated for each set and the deviation from the background matched sets was calculated as a *z* score. *P* values were calculated by direct observation of the number of background matched SNPs sets that exceeded the overlap of the GWAS index SNP set (minimum possible uncorrected *P* value is therefore 1 × 10^− 4^).

### De novo motif analysis and assignment to open chromatin regions

Macrophage DHSs for the 16 data sets from the BLUEPRINT Project were merged to form a consolidated data set using BEDTools, run with default parameters. Microglia ATAC peaks for the 12 donors were similarly merged to form a consolidated set. The consolidated sets were then used as input for *de novo* motif discovery using HOMER, [23] with default parameters. The resulting motifs were then assigned to OCRs using the HOMER command findMotifs.pl with the ‘-find’ option enabled.

### Partitioned heritability using LDscore regression

LDscore regression [11, 12] was used to partition AD genetic heritability by motif-containing sites identified as being enriched at genome-wide significant loci (e.g. CEBPA, EGR1, MEF2A and SPI1 for macrophages), following the previously described methodology [12]. AD genome-wide associated results were downloaded from http://web.pasteur-lille.fr/en/recherche/u744/igap/igap_download.php, and only phase 1 data were used. The no-motif-containing set was included as a negative control. Sites were extended by ±500 base pairs, consistent with previous partitioning heritability studies [12]. LDscore files were made for each specific annotation of interest using the open source software available here: https://github.com/bulik/ldsc/wiki. The MHC region (chr6:26,000–34,000 kb) and APOE region (chr19:44,400–46,500 kb) were removed. The results remain significant with the inclusion of these regions (data not shown). Each annotation was added to the baseline model independently, creating five separate models. The baseline model includes 24 non-cell-specific annotations that cover a range of DNA features, such as coding, 3' untranslated region, promoter, intronic, H3K4me1 marks, H3K4me3 marks, H3K9ac marks, H3K27ac marks, DNase I hypersensitivity sites, chromHMM and Segway predictions, regions conserved in mammals, super-enhancers and FANTOM5 enhancers (please see Finucane et al. [12] for more information about the baseline model).

### Web resources

*Software:*

LDscore: https://github.com/bulik/ldsc/wiki

HOMER: http://homer.ucsd.edu/homer/motif/index.html

SNPsnap: https://data.broadinstitute.org/mpg/snpsnap/

BEDTools: http://bedtools.readthedocs.io/en/latest/

*Data availability:*

Data generated by the Roadmap Epigenomics Project were downloaded from http://egg2.wustl.edu/roadmap/data/byFileType/peaks/consolidated/broadPeak/DNase/

http://egg2.wustl.edu/roadmap/data/byFileType/peaks/consolidated/broadPeak/

http://egg2.wustl.edu/roadmap/data/byFileType/peaks/consolidated/narrowPeak/

Blueprint monocyte and macrophage DHSs were downloaded from http://ftp.ebi.ac.uk/pub/databases/blueprint/data/homo_sapiens/GRCh37/

AD genome-wide associated results were downloaded from http://web.pasteur-lille.fr/en/recherche/u744/igap/igap_download.php

1000 Genomes data were downloaded from http://www.internationalgenome.org/about#ProjectSamples

Microglia ATAC-seq data were obtained from: https://www.ncbi.nlm.nih.gov/projects/gap/cgi-bin/study.cgi?study_id=phs001373.v1.p1

## Results

### Enrichment of AD risk variants at DNase hypersensitivity sites across tissue/cell types

**Fig. 1:**
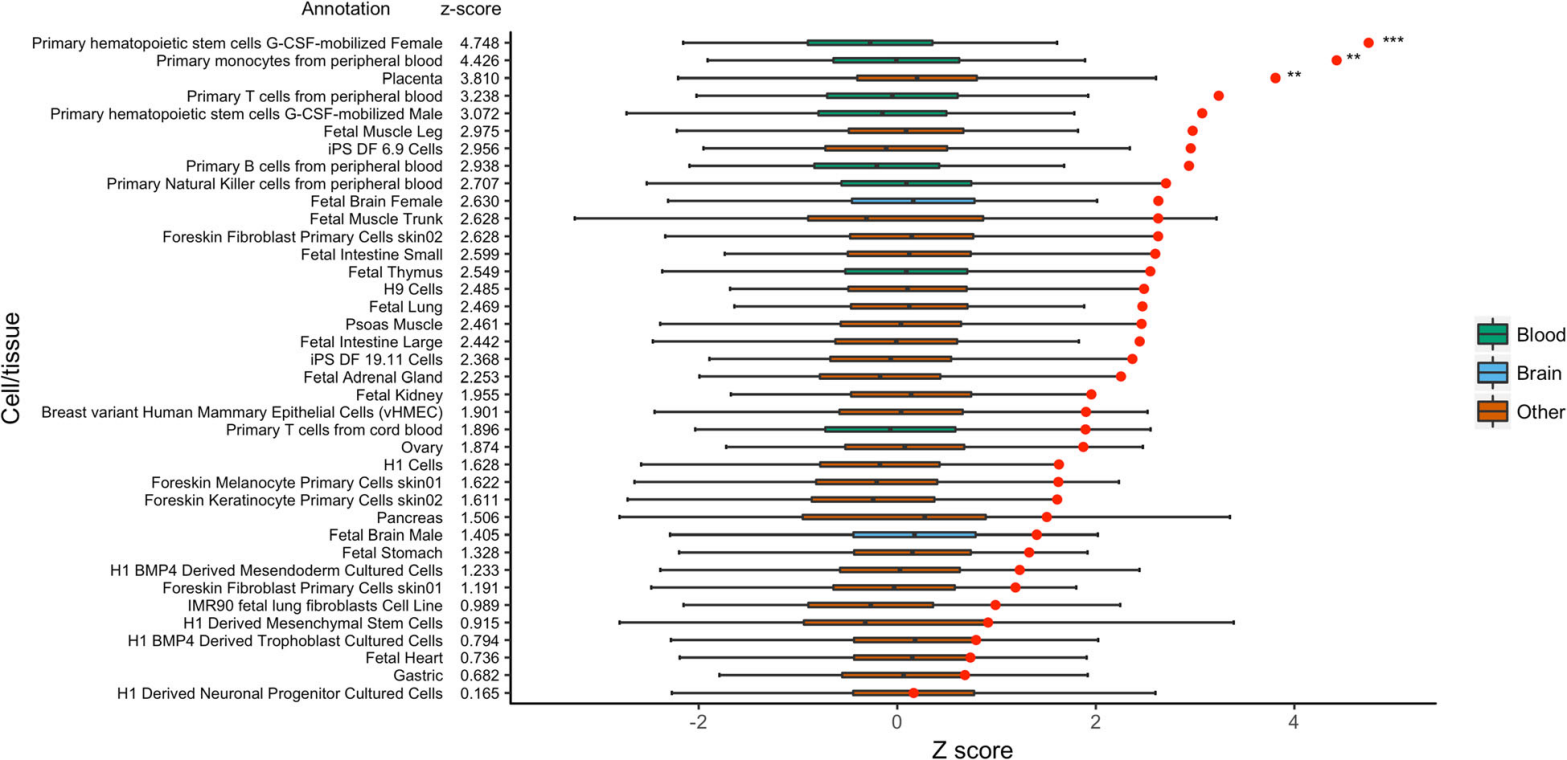
Overlap between genome-wide significant AD risk variants and DNase hypersensitivity sites from 38 tissues profiled by the Roadmap Epigenomics Project. AD risk variants and 10,000 sets of matched SNPs were intersected with DNase hypersensitivity sites. *Z* scores were calculated for the AD risk variants set for each tissue type. The *x*-axis is the *z* score and the *y*-axis the tissue types. Box plots indicate the distribution of overlap from the 10,000 background matched SNP sets. Tissue have been coded as blood (green), brain (blue) and other (orange). Red circles are the *z* scores for the AD risk variants set. *P* values were calculated from the observed overlap of the 10,000 background matched SNP sets. *P* values are corrected using the method described by Benjamini and Hochberg [24]. AD Alzheimer’s disease, SNP single nucleotide polymorphism. ***P < 0.005, **P < 0.01

We assessed whether AD risk variants (index SNPs and variants in LD at *r*^2^ > 0.8) were preferentially located at DHSs from a panel of 38 tissues profiled by the Roadmap Epigenomics Consortium [7]. Three cell/tissue types remained significant after correcting for all tests of enrichment (DNase and the three histone modifications) using the method described by Benjamini and Hochberg [24] (Fig. 1). Of these, two were immune cell types (primary haematopoietic stem cells G-CSF-mobilised, *z* score = 4.75, corrected *P* = 4.2 × 10^− 3^; and primary monocytes from peripheral blood, *z* score 4.43, corrected *P* = 5.9 × 10^− 3^). Several other immune cell types ranked highly in the analysis with four of the five most enriched tissue types being immune cells. However, these did not remain significant after correction for multiple testing. Only two brain samples, both foetal, were available in this DHS data set, and neither showed significant enrichment after correction for multiple testing (*z* score = 2.63 and 1.40, uncorrected *P* = 0.011 and 0.140). Full details of the results for each sample can be found in Additional file 1: Table S1. To confirm our cell/tissue-type enrichments, we also performed enrichment analyses using regions marked by the histone modifications H3K27ac, H3K4me1 and H4K3me3. For all three histone modifications, the largest enrichment was observed in monocytes (Additional file 2: Table S2, Additional file 3: Table S3 and Additional file 4: Table S4). Although several immune cell types were significantly enriched across these analyses, only monocytes were significant in all four chromatin feature analyses.

### Enrichment of AD risk variants at DNase hypersensitive sites in monocytes and macrophages

**Fig. 2:**
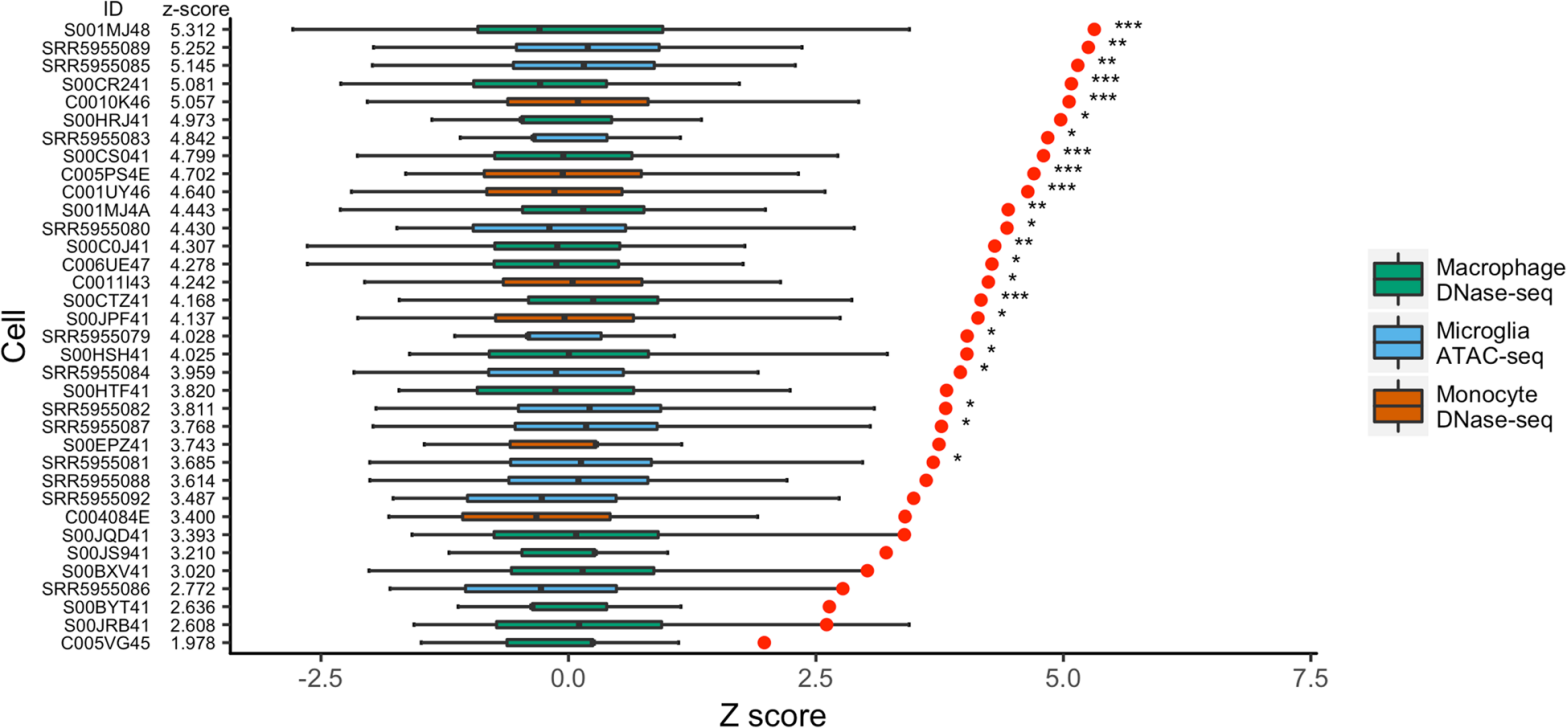
Overlap between genome-wide significant AD risk variants and open chromatin sites identified in monocyte, macrophage and microglia samples. AD risk variants and 10,000 sets of matched SNPs were intersected with open chromatin regions. *z* scores were calculated for the AD risk variants set for each tissue type. The *x*-axis is the *z* score and the *y*-axis the cell type. Box plots indicate the distribution of overlap from the 10,000 background matched SNP sets. Red circles are the *z* scores for the AD risk variants set. *P* values were calculated from the observed overlap of the 10,000 background matched SNP sets. *P* values have been corrected for 37 tests. AD Alzheimer’s disease, SNP single-nucleotide polymorphism. ***P < 0.005, **P < 0.01, *P < 0.05

Given that data generated from the Roadmap Epigenomics Consortium are derived from a limited number of donors, we sought to replicate these findings and test additional immune cell types. DNase hypersensitivity data from 16 macrophage and seven monocyte samples were available from the Blueprint Epigenome Project (http://dcc.blueprint-epigenome.eu/#/home). Using these data, enrichment *z* scores for the overlap with AD risk variants ranged from 3.00 to 5.07 (mean = 4.12) for the seven monocyte samples, and 1.98 to 5.32 (mean = 3.88) for the 16 macrophage samples (Fig. 2). In total, 14 of the 23 samples tested were significant after correction for multiple testing using the Bonferroni method to correct for 37 tests (35 monocyte/macrophage/microglia samples plus the two consolidated sets), replicating the enrichment of AD variants at immune cell DHSs, and identifying macrophages as a potential cell type affected by AD genetic risk.

**Fig. 3:**
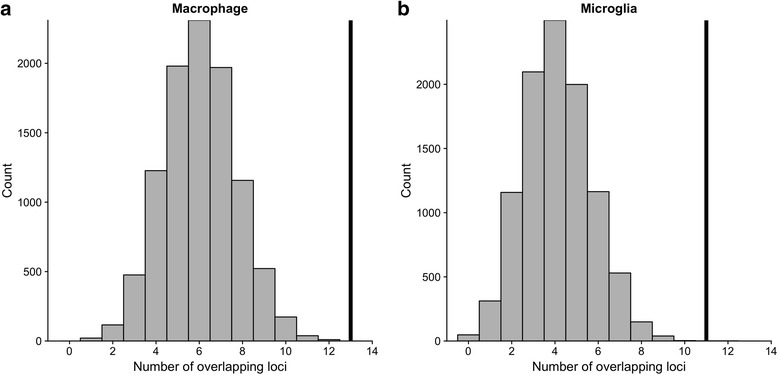
Overlap between genome-wide significant AD risk variants and open chromatin regions from the consolidated set of (**a**) macrophage and (**b**) microglia samples. Grey histogram bars are the distribution of overlap from the 10,000 background matched SNP sets. The vertical black line is the number of overlapping loci from the AD risk variants set. AD Alzheimer’s disease, SNP single-nucleotide polymorphism

To reduce inconsistencies arising from selecting individual donor samples, a consolidated set of macrophage DHSs was generated by merging the peaks from the 16 different data files. AD risk variants were similarly enriched at DHSs in this consolidated set (*z* score = 4.10, *P* < 1 × 10^− 4^, corrected *P* < 3.7 × 10^− 3^), with 13 of the 18 loci tested having at least one overlapping SNP (Fig. 3). At these 13 loci, the number of SNPs overlapping macrophage DHSs ranges from 1 to 11 (Additional file 5: Table S5 and Additional file 6: Figure S1), indicating multiple potential causal alleles. These loci contain genes with both overt immune cell functions (e.g. *INPP5D*) and no known immune-cell-specific activity (e.g. *BIN1* and *PICALM*).

### Enrichment of AD risk variants at open chromatin regions in microglia

We obtained publicly available human microglia open chromatin (ATAC-seq) data from 12 donors [18] to investigate the role of the resident brain macrophage in AD genetic risk mechanisms. We observed enrichment *z* scores ranging from 2.77 to 5.25 (mean = 4.07). In total, nine donor samples were significant after Bonferroni correction for the 37 tests (35 monocyte/macrophage/microglia samples plus the two consolidated sets). AD risk variants were also enriched at microglia ATAC-seq peaks using the consolidated peak set (*z* score = 4.34, corrected *P* = 0.011), with a total of 11 loci containing at least one SNP that overlapped an ATAC-seq peak (Fig. 3). Additional file 7: Table S6 contains a full list of overlapping SNPs and gene annotations.

### Enrichment of AD risk SNPs at open chromatin regions containing specific transcription factor motifs

We further investigated the localisation of AD risk variants to specific subsets of macrophage and microglia OCRs defined by the presence of specific transcription factor DNA binding motifs. *De novo* motif analysis of the consolidated sets of macrophage DHS or microglia ATAC-seq peaks was performed using HOMER [23].

**Fig. 4:**
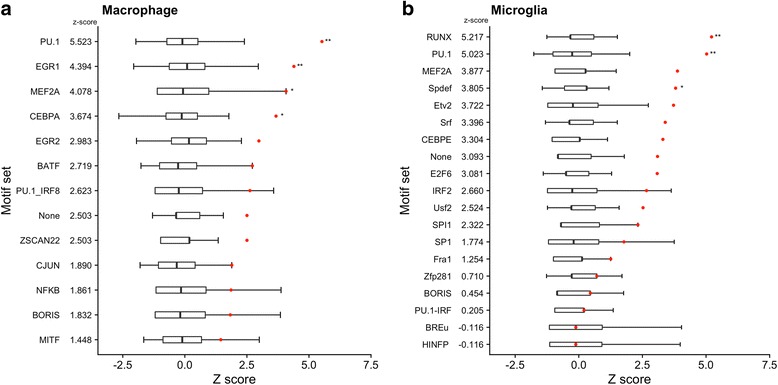
Overlap between genome-wide significant AD risk variants and transcription factor motif-containing open chromatin sites from the consolidated macrophage (**a**) and microglia (**b**) data. AD risk variants and 10,000 sets of matched SNPs were intersected with transcription factor motif-containing open chromatin region sets and one no-motif-containing set for each cell type. The *x*-axis is the *z* score and the *y*-axis is the transcription factor motif. Box plots indicate the distribution of overlap from the 10,000 background matched SNP sets. Red circles are the *z* scores for the AD risk variant set. *P* values were calculated from the observed overlap of the 10,000 background matched sets. *P* values have been adjusted within each cell type using the Bonferroni correction for the number of motif sets tested. AD Alzheimer’s disease, SNP single-nucleotide polymorphism. ***P < 0.005, *P < 0.05

In the macrophage DHS, this identified 15 enriched motifs (Additional file 8: Table S7), including established regulators of immune cell function (e.g. SPI1 and NFKB). We then grouped DHSs according to the presence or absence of a motif for each of the 15 motifs identified, generating 16 subsets, one for each specific transcription factor motif and one with DHSs that lacked any of these motifs. Two motif sets were removed from the analysis as fewer than 1000 of the 10,000 background matched SNPs showed any overlap. AD risk variants were significantly enriched after correction for multiple testing using the Bonferroni method at DHSs containing the motifs SPI1 (PU.1) (*z* score = 5.53, corrected *P* < 1.30 × 10^− 3^), EGR1 (*z* score = 4.40, corrected *P* < 1.30 × 10^− 3^), MEF2A (*z* score = 4.08, corrected *P* = 0.023) or CEBPA (*z* score = 3.68, corrected *P* = 0.013) (Fig. 4a). The SPI1 (PU.1) motif set captured all 13 of the loci that showed an overlap with the consolidated macrophage DHS set. The number of SNPs overlapping the SPI1 motif-containing DHS at each locus ranged from 1 to 7, implicating multiple potential causal SNPs.

*De novo* motif analysis of the microglia ATAC-seq peaks identified 19 motifs (Additional file 9: Table S8), including known lineage-determining factors such as SPI1 and IRF [25]. Sets of motif-containing peaks were then generated as described above. After intersecting with SNP data, one motif set was removed from the analysis as fewer than 1000 of the 10,000 background matched SNPs showed any overlap. AD risk variants were significantly enriched at ATAC-seq peaks containing motifs for RUNX (*z* score = 5.22, corrected *P* < 1.9 × 10^− 3^), SPI1 (PU.1) (*z* score = 5.02, corrected *P* < 1.9 × 10^− 3^) and Spdef (*z* score = 3.80, corrected *P* = 0.027) after correction for multiple testing using the Bonferroni method (Fig. 4b). Like the macrophages, the SPI1 (PU.1) set captured the largest number of loci, accounting for nine of the 11 overlaps identified using all ATAC-seq peaks in the consolidated set. We validated our motif-based findings using SPI1 (PU.1) ChIP-seq data from human microglia [18]. AD risk variants were significantly enriched at these experimentally identified SPI1 (PU.1) bound regions (*z* score = 4.62, *P* = 2 × 10^− 4^; Additional file 6: Figure S2).

For both macrophages and microglia, SPI1 (PU.1) motif-containing OCRs were significantly enriched, indicating that this class of OCRs is of relevance to AD genetic risk mechanisms in both cell types. CEBP and MEF2 motif-containing OCRs survived correction for multiple testing in macrophages and were nominally significant (uncorrected *P* < 0.05) in microglia. Several motif-containing OCR sets were tested for only one cell type as *de novo* motif analysis did not identify them in the other, e.g. EGR1 for macrophages and RUNX for microglia.

### Common variant heritability of AD is enriched at specific transcription factor motif-containing open chromatin regions

Although many genome-wide significant AD risk loci have been identified, they account for a small proportion of the genetic heritability. Instead, thousands of variants across the entire genome collectively contribute to the polygenic inheritance of AD. We reasoned that transcription factor motif-containing OCRs identified as being enriched at genome-wide significant loci would also be important for mediating polygenic inheritance. Therefore, we partitioned AD heritability by macrophage or microglia motif sets using LDscore regression [12].

Consistent with the macrophage SNP enrichment analysis of genome-wide significant loci, AD heritability was significantly enriched at variants in the DHS motif sets SPI1 (PU.1) (enrichment = 8.93, corrected enrichment *P* = 0.012), MEF2A (enrichment = 19.22, corrected enrichment *P* = 0.022), CEBPA (enrichment = 9.72, corrected enrichment *P* = 3.43 × 10^− 3^) and EGR1 (enrichment = 14.48, corrected enrichment *P* = 5.14 × 10^− 4^). *P* values for all the transcription factors tested withstood Bonferroni correction for multiple testing (Table 1). Importantly, the no-motif DHS set was not significantly enriched (corrected enrichment *P* = 0.625) (Table 1). Additional file 10: Table S9 contains the full results.

**Table 1:** Enrichment of AD heritability at variants within EGR1, CEBPA, MEF2A, SPI1 and no-motif-containing macrophage DNase hypersensitivity sites

DHS motif set	Enrichment	Enrichment *P* value	Corrected *P* value
EGR1	14.481	1.03 × 10^− 4^	5.14 × 10^− 4^
CEBPA	9.716	6.86 × 10^− 4^	3.43 × 10^−3^
SPI1	8.933	2.43 × 10^−3^	0.012
MEF2A	19.222	4.46 × 10^−3^	0.022
No-motif	7.661	0.125	0.623

AD heritability was partitioned by transcription factor motif-containing DNase hypersensitivity sites using LDscore regression [12]. AD heritability was significantly enriched (after correcting for multiple testing) at all four transcription factor motif-containing DNase hypersensitivity sites but not the no-motif set

*AD* Alzheimer’s disease

In microglia, AD heritability was significantly enriched at variants in the OCR motif sets SPI1 (PU.1) (enrichment = 16.28, corrected enrichment *P* = 4.39 × 10^− 3^) and Spdef (enrichment = 19.92, corrected enrichment *P* = 0.040). The RUNX OCR motif set was not significantly enriched (enrichment = 14.09, correct enrichment *P* = 0.412), nor was the no-motif set (enrichment = 20.27, corrected enrichment *P* = 0.168). *P* values were corrected using Bonferroni correction for multiple testing accounting for the number of tests undertaken within each cell type (Table 2). Additional file 11: Table S10 contains the full results. The enrichment of AD heritability at variants in SPI1 motif-containing OCRs was validated using the SPI1 ChIP-seq data. Variants at these SPI1 bound regions were also substantially enriched for AD heritability (enrichment = 20.56, enrichment *P* = 6.9 × 10^–4^).

**Table 2:** Enrichment of AD heritability at variants within SPI1, Spdef, RUNX and no-motif-containing microglia ATAC-seq peaks

Motif set	Enrichment	Enrichment *P* value	Corrected *P* value
SPI1	16.28	1.10 × 10^−3^	4.39 × 10^− 3^
Spdef	19.92	9.93 × 10^− 3^	0.040
No-motif	20.27	0.042	0.168
RUNX	14.09	0.103	0.412

AD heritability was partitioned by transcription factor motif-containing ATAC-seq peaks using LDscore regression [12]. AD heritability was significantly enriched (after correcting for multiple testing) at SPI1 and Spdef transcription factor motif-containing ATAC-seq peaks but not the RUNX or no motif sets

*AD* Alzheimer’s disease

## Discussion

Although GWAS have identified thousands of variants that influence diseases and traits, the majority are located in non-coding regions of the genome [2]. Combined with small effect sizes, the biological interpretation of these results is challenging. We have integrated results from GWAS of AD with OCRs identified in different tissue types, first by using genome-wide significant loci and then extending our analyses to genome-wide measurements of partitioned heritability. Through this two-stage approach, we identify alleles of potential functional significance that are amenable to further mechanistic investigation, and show variants contributing to polygenic inheritance are likely to operate through shared mechanisms. Specifically, these analyses identified macrophage and microglia transcriptional networks in which both genome-wide significant alleles and polygenic risk for AD are enriched.

The localisation of AD risk variants to DHSs from multiple immune cell types assayed by the Epigenomics Roadmap Project highlights their potential importance in mediating the effects of AD genetic risk, and is in agreement with other studies [4, 5]. Our analyses using histone modifications that are indicative of active gene regulatory elements, also strongly supports the role of immune cells, particularly monocytes, in AD genetic risk mechanisms. Enrichment at all three histone modifications tested suggests risk mechanisms involve multiple types of regulatory elements (e.g. promoters and enhancers). Combined with results generated using data from the Blueprint Epigenome Project, we provide replicated evidence for the enrichment of AD risk variants at monocyte DHSs. A similar enrichment is also observed at macrophage DHSs, a cell type derived from monocytes that have invaded a target tissue. Finally, we show that microglia are also plausibly linked to AD genetic risk mechanisms via regions of open chromatin. Unlike studies of post-mortem material, where cause cannot easily be separated from consequence, genetic associations do not suffer from problems of reverse causation. Therefore, our findings implicate immune cell dysfunction as a causal factor in AD risk. Given the extensive overlap between regulatory elements in related cell types, it is not currently possible to identify a single causal immune cell type and we cannot exclude the involvement of multiple cell types in AD risk mechanisms. However, the location of microglia in the brain positions them as the likely causal candidates.

In contrast to the significant enrichment at immune cell DHSs, AD risk variants were not enriched at brain DHSs identified using bulk tissue. However, DHSs data from the Epigenomics Roadmap Project contains only two brain samples, both foetal. The enrichment at microglial OCRs suggests that they are the plausible brain cell type in which AD risk mechanisms operate, and that profiles from bulk tissue suffer from lack of cell-type specificity. It is necessary to investigate additional brain data as they become available, particularly those that can resolve cell-type specific information [26]. It should also be noted that the currently available data are primarily derived from healthy donors under basal conditions. To investigate the gene regulatory mechanisms underlying genetic disease risk fully, it may be necessary to investigate cells under a variety of conditions, including those thought to be environmental risk factors for disease.

Consistent with gene-based pathway analysis of AD GWAS [27, 28], these loci harbour genes such as *PTK2B* and *INPP5D* that encode for proteins with recognised immune functions and have immune cell-type enriched expression. However, it is at the level of DNA regulatory elements that tissue-specific risk mechanisms are generated. Indeed, our analysis identifies a number of ubiquitously expressed genes (e.g. *BIN1* and *CD2AP*) at which AD associated risk variation could credibly operate in immune cells, including microglia. Therefore, the number of AD risk loci that impact on immune cell function is likely to be larger than that captured by current gene-based pathway annotation methods. It is now important to identify the biological processes that are disrupted by AD risk variants in immune cells.

Of the 18 genome-wide significant loci tested, 13 have at least one variant located in a macrophage DHS and 11 in a microglial OCR, indicating that the majority of AD risk loci plausibly operate to alter gene expression in these cells. At most of these loci, more than one SNP overlapped an OCR, suggesting that individual risk loci are likely to harbour multiple functional variants. By focusing on OCRs containing transcription factor motifs, the number of overlapping SNPs at each locus is reduced. For example, in microglia, eight of the 11 loci contain three or fewer SNPs overlapping a SPI1 motif-containing OCR. These variants can, therefore, be prioritised for further molecular characterisation.

Having established an enrichment of AD risk variants at macrophage and microglia OCRs, we investigated their localisation to OCRs containing motifs for specific transcription factors. Within a given cell type, thousands of transcriptional regulators contribute to the control of gene expression, but master regulators, often cell type specific, can be recovered by a motif analysis of regulatory element sequences. In both macrophage and microglia, AD risk variants were enriched at OCRs containing specific transcription factor motifs, supporting the hypothesis that risk variants are localised to specific transcription factor targeted OCRs, including experimentally determined SPI1 bound regions in microglia.

Of particular interest is the enrichment of AD risk SNPs at SPI1 and MEF2A motif-containing OCRs. Genetic variants at, or in close proximity to, *SPI1* and *MEF2C* (HOMER reports that the MEF2C and MEF2A motifs have a similarity score of 0.94) have been identified as significant AD risk loci [1, 29]. Impaired transcriptional control by these factors, either through altered gene expression in *cis* or via disrupted DNA binding due to genetic variants at target sites, is likely to play a central role in AD genetic risk mechanisms. The importance of variants in these motif-containing OCRs extends beyond those reaching genome-wide significance, providing evidence that the thousands of subthreshold variants contributing to polygenic risk collectively operate by similar mechanisms. Although enrichment at these sites is large (~9–19 fold), and account for a substantial proportion of the total SNP-chip heritability, the *P* values reported are weaker than those observed in analyses of some other diseases using chromatin features [12]. This is most likely due to the low SNP-chip heritability of AD as calculated by LDscore regression (~7%, http://ldsc.broadinstitute.org/lookup/). GWAS data from larger cohorts will be important for defining risk mechanisms at increased molecular resolution. Similarly, the identification of transcription factor motifs from studies of open chromatin derived from additional methods will reduce potential single source biases.

More generally, our results support a model of polygenic disease risk that is enriched at defined transcriptional networks operating in cell types relevant to disease. For other complex disorders such as type 2 diabetes, genome-wide significant risk variants have been shown to localise to specific transcription factor binding sites in islet cells [30, 31], but the extent to which variants in these binding sites contribute to polygenic inheritance was not investigated. We show that polygenic risk arising from non-coding variation is localised to specific transcription factor networks. For AD, this is most prominent for a potential SPI1-driven network, consistent with a targeted investigation [32].

*SPI1* encodes a transcription factor known to be critical for the development and function of haematopoietic cell lineages [33], including microglia [25]. Decreased expression of *SPI1* and *CEBPA* (also identified through motif enrichment analysis in macrophages) is observed after a reduction in AD-like pathology and behaviour in APPswe/PSEN1dE9 mice following pharmacological inhibition of the receptor CSF1R [34]. Therefore, our results link polygenic AD risk mechanisms to transcriptional networks that have therapeutic validity. The identification of upstream regulators of these transcription factors may yield novel targets that are important for AD therapies.

## Conclusions

In summary, integration of GWAS results with sites of open chromatin identifies immune cells as likely mediators of common variant genetic risk for AD. The majority of genome-wide significant AD risk loci plausibly operate in peripheral monocytes, macrophages and/or microglia, and we identify candidate SNPs at these loci suitable for targeted mechanistic studies based on shared OCR annotations. Within open chromatin sites, those containing specific DNA motifs drive this enrichment. Similarly, genetic variants at these sites capture a substantial proportion of the AD common variant SNP-chip heritability, ~67% for the SPI1 targeted sites, increasing the molecular resolution of AD genetic risk mechanisms from cell type to transcriptional networks. We provide evidence for the causal role of microglia in AD pathogenesis and therefore, a parsimonious explanation for the involvement of immune cells in AD risk mechanisms. Furthermore, we establish that the thousands of variants contributing to AD polygenic risk are enriched at specific macrophage/microglial transcriptional networks, placing them in tangible biological pathways amenable to future mechanistic studies.

## Additional files

Additional file 1: **Table S1.** Enrichment of AD-risk variants at DHSs from Roadmap Epigenomics Consortium data. (XLSX 36 kb)

Additional file 2: **Table S2.** Enrichment of AD-risk variants at H3K27ac peaks from Roadmap Epigenomics Consortium data. (XLSX 54 kb)

Additional file 3: **Table S3.** Enrichment of AD-risk variants at H3K4me1 peaks from Roadmap Epigenomics Consortium data. (XLSX 56 kb)

Additional file 4: **Table S4.** Enrichment of AD-risk variants at H4K3me3 peaks from Roadmap Epigenomics Consortium data. (XLSX 48 kb)

Additional file 5: **Table S5.** AD-risk SNPs overlapping macrophage DHS. (XLSX 10 kb)

Additional file 6: **Figure S1.** Open chromatin regions (OCRs) identified in macrophages and microglia at Alzheimer’s disease risk loci containing the genes *BIN1* and *CASS4*. **Figure S2.** Overlap between Alzheimer’s disease risk loci and genomic regions bound by SPI1 in human ex vivo microglia. (PDF 595 kb)

Additional file 7: **Table S6.** AD-risk SNPs that overlap microglia ATAC-seq peaks. (XLSX 12 kb)

Additional file 8: **Table S7.** Results from the *de novo* motif analysis performed using HOMER on the macrophage DHS. (XLSX 10 kb)

Additional file 9: **Table S8.** Results from the *de novo* motif analysis performed using HOMER on microglia ATAC-seq peaks. (XLSX 10 kb)

Additional file 10: **Table S9.** Enrichment of AD heritability at variants within the motif-containing macrophage DHS. (XLSX 9 kb)

Additional file 11: **Table S10.** Enrichment of AD heritability at variants within motifs containing microglia ATAC-seq peaks. (XLSX 30 kb)

## Abbreviations

AD: Alzheimer’s disease
ATAC-seq: Assay for transposase-accessible chromatin using sequencing
ChIP: Chromatin immunoprecipitation
DHS: DNase hypersensitivity site
GWAS: Genome-wide association studies
LD: Linkage disequilibrium
MHC: Major histocompatibility complex
OCR: Open chromatin region
SNP: Single nucleotide polymorphism
