# Supplementary material for: Genetic risk for Alzheimer’s disease is concentrated in specific macrophage and microglial transcriptional networks
Source: Genome Med. 2018 Feb 26;10:14. doi: 10.1186/s13073-018-0523-8 (PMC5828245; doi:10.1186/s13073-018-0523-8)
Supplement: Supplementary file 6 — Figure S1. Open chromatin regions (OCRs) identified in macrophages and microglia at Alzheimer’s disease risk loci containing the genes BIN1 and CASS4. Figure S2. Overlap between Alzheimer’s disease risk loci and genomic regions bound by SPI1 in human ex vivo microglia. (PDF 595 kb) [file 13073_2018_523_MOESM6_ESM.pdf]

Supplemental Figures

Figure S1.

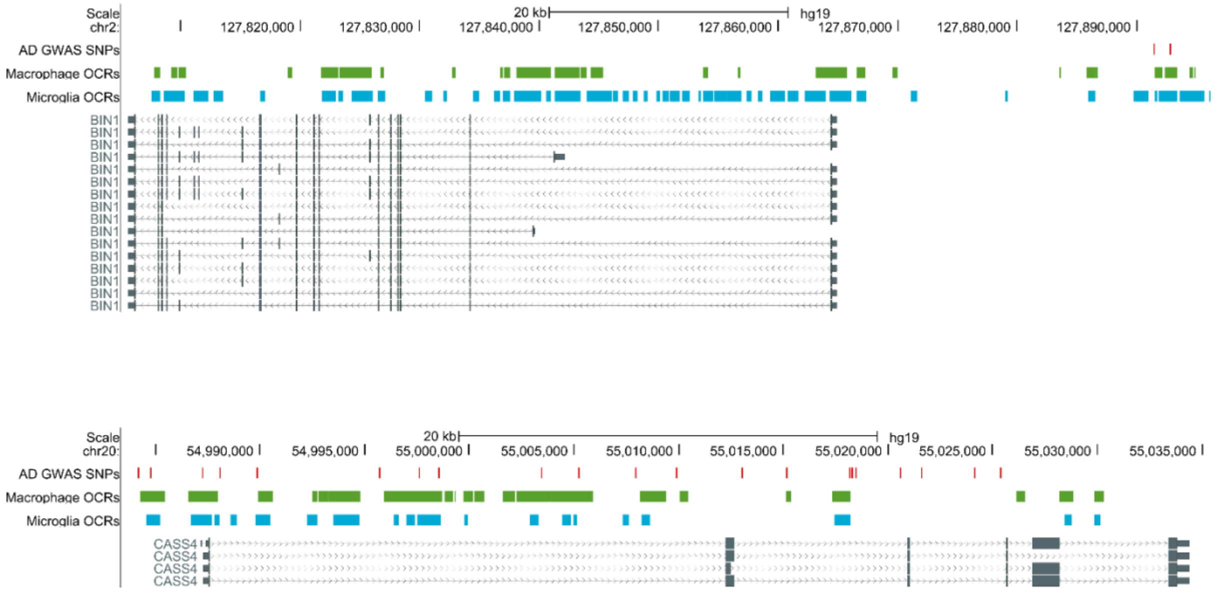

Figure S1. Open chromatin regions (OCRs) identified in macrophages and microglia at Alzheimer's disease risk loci containing the genes *BIN1* (top panel) and *CASS4* (bottom panel). Alzheimer's disease risk variants are shown in red, macrophage OCRs in green and microglia OCRs in blue.

Figure S2.

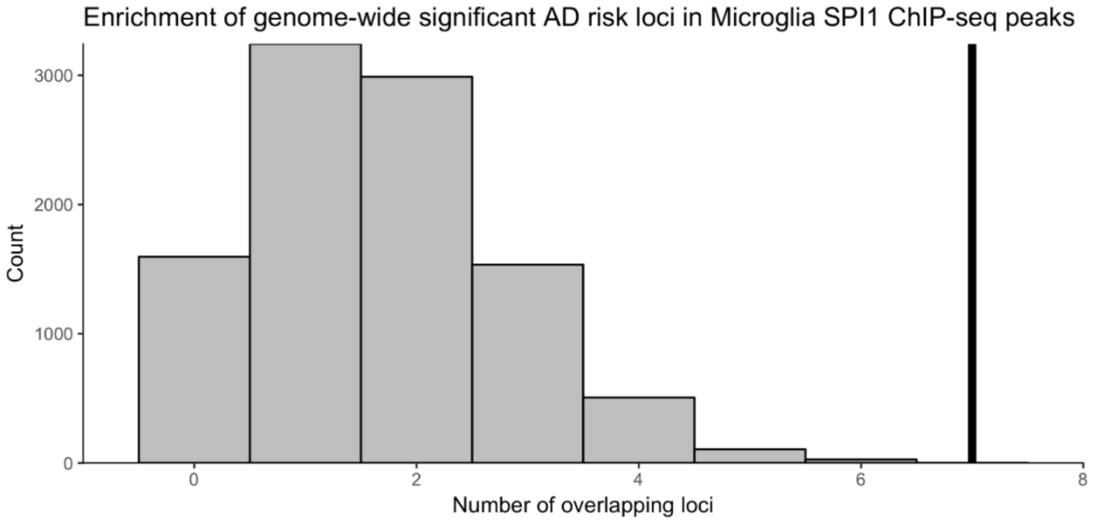

Figure S2. Overlap between Alzheimer's disease risk loci and genomic regions bound by SPI1 in human ex vivo microglia. The grey bars are the overlap at 10,000 matched SNP set loci, the solid black line is the overlap with genome-wide significant Alzheimer's disease risk loci.
